# Fermented Food and Non-Communicable Chronic Diseases: A Review

**DOI:** 10.3390/nu10040448

**Published:** 2018-04-04

**Authors:** Doreen Gille, Alexandra Schmid, Barbara Walther, Guy Vergères

**Affiliations:** 1 Agroscope, Schwarzenburgstrasse 161, 3003 Berne, Switzerland; doreen.gille@uzh.ch (D.G.); alexandra.schmid@agroscope.admin.ch (A.S.); barbara.walther@agroscope.admin.ch (B.W.); 2 Epidemiology, Biostatistics and Prevention Institute, University of Zurich, 8001 Zurich, Switzerland

**Keywords:** fermented food, dairy, plants, cardiometabolic health, cancer, type 2 diabetes, meta-analysis, randomized controlled trial

## Abstract

Fermented foods represent a significant fraction of human diets. Although their impact on health is positively perceived, an objective evaluation is still missing. We have, therefore, reviewed meta-analyses of randomized controlled trials (RCT) investigating the relationship between fermented foods and non-transmissible chronic diseases. Overall, after summarizing 25 prospective studies on dairy products, the association of fermented dairy with cancer was found to be neutral, whereas it was weakly beneficial, though inconsistent, for specific aspects of cardio-metabolic health, in particular stroke and cheese intake. The strongest evidence for a beneficial effect was for yoghurt on risk factors of type 2 diabetes. Although mechanisms explaining this association have not been validated, an increased bioavailability of insulinotropic amino acids and peptides as well as the bacterial biosynthesis of vitamins, in particular vitamin K2, might contribute to this beneficial effect. However, the heterogeneity in the design of the studies and the investigated foods impedes a definitive assessment of these associations. The literature on fermented plants is characterized by a wealth of in vitro data, whose positive results are not corroborated in humans due to the absence of RCTs. Finally, none of the RCTs were specifically designed to address the impact of food fermentation on health. This question should be addressed in future human studies.

## 1. Introduction

### 1.1. Introduction to Fermented Foods

Fermented foods and beverages are generally defined as products made by microbial organisms and enzymatic conversions of major and minor food components. These products have been produced and consumed since the development of human civilization by all cultures around the world covering all types of food groups, including dairy, vegetables, legumes, cereals, starchy roots and fruits as well as meat and fish. Basically, fermentative processes can be categorized by the microorganisms and primary metabolites involved: alcohol and carbon dioxide (yeast), acetic acid (*Acetobacter*), lactic acid (lactic acid bacteria (LAB)), propionic acid (*Propionibacterium*), and ammonia and fatty acids (*Bacillus*, molds) [1]. The reasons for fermenting foods are as simple as they are plausible: (i) improvement and extension of a product’s storage time [2], (ii) improvement of organoleptic quality [3] and (iii) improvement of a product’s nutritional properties [4]. The health-promoting potential of fermented foods has risen the awareness of this food group, resulting in suggestions to include them as part of national dietary recommendations [2,5].

Amongst fermented foods, the effects of fermented dairy products on health and disease, in particular, have been the focus of research in the past. Multiple benefits against non-communicable diseases and metabolic impairments have been attributed to this food group, such as weight maintenance, reductions in risks of cardiovascular diseases, diabetes type 2 and overall mortality, and improved glucose metabolism [6,7,8,9,10]. Also, for kimchi, a traditional Korean vegetable mix, scientific evidence indicates anti-diabetic and anti-obesity potential [11]. For other public health relevant diseases concerning mood and brain activity [12,13,14] as well as immune-related pathologies (e.g., sclerosis and arthritis) [15], beneficial effects of fermented foods have been proposed but will need further investigation, particularly in the form of clinical studies.

The proposed modes of action that make fermented foods attractive for consumption regarding potential health effects are manifold. One example is the simple, but effective, way of helping lactose intolerant persons to digest lactose by utilizing microbial organisms and their galactosidases that start to cleave the milk sugar during fermentation and continue with this activity in the gastrointestinal tract (GIT) of humans. This process allows affected people to ingest dairy products (cheese and yoghurt) without experiencing any symptoms of lactose intolerance [4]. Another example is the proteolytic activity of LAB in milk and other foods that can result in increased concentrations of bioactive peptides [16].

Fermentation can also lead to the enrichment of new compounds in food products, such as some B vitamins, including folate, riboflavin, B12, as well as vitamin K2 (menaquinones), which are synthesized from various non-vitamin precursors by certain bacteria in plants and dairy foods [17,18]. Lastly, fermented foods are also ideal vehicles for the delivery of microbes/probiotics to the human GIT. Fermentation-associated microorganisms might alter the intestinal composition or function of the autochthonous microbiota in the GIT. However, the magnitude of these changes and importance in probiotic efficacy is currently a point of contention [19,20,21].

### 1.2. Fermented Foods in Food Guides

Despite the many potential benefits of fermented foods, their recommended consumption has not been widely translated to global inclusion in food guides.

The food guides of Canada and the USA recommend the intake of yoghurt and kefir as items listed under the dairy products section, but, in general, there is no inclusion of fermented products as a healthy food category. Japan highlights the importance of consuming foods from every food group in order to achieve a well-balanced diet but does not specifically mention fermented foods. Almost the same pattern appears in the Chinese recommendations with the exception that the Chinese Nutrition Society highlights the use of yoghurt for people suffering from lactose malabsorption. Fermented foods have a long history in Asia, where the earliest records of fermenting processes can be traced back to 300 BC in China, and the third century in Japan [22]. China is also said to be the birth place of fermented vegetables and is a pioneer in the use of mold to make food. Considering the long tradition and high importance in the Asian diet, it is surprising that food guides in Japan and China do not explicitly recommend them as a category. One exception in Asia is the Indian food guide, which stresses the consumption of fermented foods for the public and specifically, for pregnant women (“eat more whole grains, sprouted grams and fermented foods”). The National Institute of Nutrition’s 2010 “Dietary Guidelines for Indians” particularly describes the enhanced digestibility of fermented foods as well as their increased nutritional value due to an increased production of vitamins (B and C) [2].

Also, European food guides lack recommendations for fermented foods. For example, the UK puts emphasis on carbohydrates, fruits and vegetables but does not mention fermented foods as a special category. The Swedish model for healthy eating has no particular section regarding dairy products or any other fermented food.

However, the consumption of fermented milks, in particular, is very common in Europe. Fermented milks have been found to be beneficial for lactose maldigesting persons [4] and are thus interesting in terms of health claims. In this area, only one claim for beneficial microbes has been approved in the European Union (EU) (yoghurt improves lactose tolerance) [23]. Smug et al. [23] searched for health messages being approved by non-commercial government bodies that include fermented milks or probiotics in the nutrition guidelines in thirteen EU countries, including Switzerland. This analysis revealed that five EU member states include either probiotics or fermented milks with live bacteria in their national nutrition guidelines or recommendations (Table 1). This finding shows that some EU countries acknowledge the health benefits associated with the consumption of live bacteria.

In Switzerland, the fermented products food group is not explicitly mentioned, though individual food items are—for example, cheese and yoghurt are part of the recommendation to consume three portions of dairy products per day. Also, coffee is part of the national food pyramid, though it is promoted to the public based on its capability to contribute to humans’ liquid supply, rather than its potential beneficial characteristics as a fermented food. Other fermented foods of plant origin, such as pickles and sauerkraut or fermented soya, are not explicitly mentioned in the food pyramid [24]. A few probiotic yoghurts/drinks and bacteria have received health claim approval from the Swiss Federal Office of Public Health. So far, Switzerland is the only country having approved those claims. All seven products (five of them being probiotic sour milks/yoghurts) received approval in regard to their ability to support normal digestion and improve intestinal passage as well as reduce flatulence [25]. The consumption of these products is not explicitly recommended by the Swiss food pyramid.

The aim of this review was to give an overview on the present knowledge and gaps regarding the association of fermented foods and non-communicable chronic diseases.

## 2. Materials and Methods

### 2.1. Literature Search

The PubMed database was searched at the end of March 2017, by combining various search terms for dairy foods (“milk” or ”dairy” or “dairy products” or “yoghurt” or “yogurt” or “yoghourt” or “cheese” or “cultured milk products”) with search terms for non-communicable diseases (“cardiovascular diseases” or “cardiovascular disease” or “heart disease” or “heart diseases” or “stroke” or “myocardial infarction” or “hypertension” or “blood pressure” or “diabetes” or “mortality” or “death” or “obesity” or “metabolic syndrome” or “diabetes mellitus, type 2” or “type 2 diabetes” or “cancer”). The search was limited to the last five years (January 2012–March 2017) and to human studies. Only meta-analyses and systematic reviews were taken into consideration. Search results were evaluated, and studies in children, or those that focused on single nutrients or individual microorganisms only, were excluded.

The identified references were analyzed by specifically focusing on fermented dairy products, including yoghurt, cheese, and fermented milk and comparing the outcomes of these analyses with milk (as a reference for non-fermented dairy) and total dairy (for the total contribution of fermented and non-fermented dairy). Consequently, physiological effects of fermented dairy products which are mediated by the fermentation process, as assessed by one of the clinical indications investigated in this report, should be observed with fermented milk, yoghurt and cheese but not with milk. Such effects could also be observed with total dairy intake, provided fermented products contribute significantly to the total intake of dairy products. Finally, one should note that effects observed with the consumption of cheese, but not milk, might not be uniquely attributed to the impact of milk fermentation since cheese making also involves the removal of whey.

For probiotics, the same search was conducted as on dairy products, except that the key words “devoted to dairy products” were replaced by the key word “probiotic*”(the asterisk being the PubMed truncation symbol).

For fermented foods of plant origin, the same search strategy was used that is described above, except the key words “devoted to dairy products” were replaced by the key words “plant or soy*” or “sauerkraut or olive*” or “coffee” or “wine” or “fermented plant food*”. As the outcome of this search strategy was not satisfactory, a manual search was subsequently performed.

### 2.2. Rating of the Results from the Meta-Analyses

To rate the results of meta-analyses summarizing observational studies, the characterization of an effect as ‘beneficial’ or ‘detrimental’ was based on the direction of the effect and on the statistical significance of the dataset provided by each meta-analysis. Non-significant effects were characterized as ‘neutral’. The size of the effect was qualified as ‘weak’ for a risk reduction of ≤10% and ‘moderate’ for a risk reduction >10%. For meta-analyses summarizing three or more prospective cohort studies (grade IIa), the quality of the evidence was rated as ‘moderate’ without consideration of other important parameters of relevance to an estimation of the evidence, such as the heterogeneity of the studies, the relevance of the population studied, or the qualities of the investigated foods or probiotics tested. For meta-analyses summarizing less than three prospective cohort studies or summarizing case-control studies (grade IIIa) or retrospective studies (IIIb) the evidence was rated as ‘weak’ (see also Table S1).

To rate the results of meta-analyses summarizing randomized controlled intervention studies, the characterization of an effect as ‘beneficial’ or ‘detrimental’ was based on the direction of the effect and on the statistical significance of the dataset provided by each meta-analysis. Non-significant effects were characterized as ‘neutral’. The size of the effect was qualified as ‘weak’ for a risk reduction of ≤10% and ‘moderate’ for a risk reduction >10% compared to normal range values. All meta-analyses were summarizing three or more controlled randomized trials (grade Ia), and the quality of the evidence was therefore rated as ‘moderate’ without consideration of other important parameters of relevance to an estimation of the evidence, such as the heterogeneity of the studies, the relevance of the population studied, or the qualities of the investigated foods or probiotics tested.

The data from all meta-analyses presented in this report are summarized in Table S2 (Dairy and cardio-metabolism), Table S3 (Dairy and cancer) and Table S4 (Probiotics and cardio-metabolism). After reviewing the results, we decided to exclude probiotics because not enough information was available to differentiate between the different strains or to find any clear associations between specific strains and non-communicable diseases.

This report does not focus on particular brands of products (e.g., Swiss cheese), particular bacterial strains, probiotic products, or nutraceuticals. As such, the products described in this report (e.g., cheese or yoghurt) are to be considered from a generic point of view.

## 3. Results

### 3.1. Scientific Evidence on Fermented Dairy Products

#### 3.1.1. Meta-Analyses from Prospective Studies and Randomized Controlled Trials (RCTs) on Cardio-Metabolic Diseases

To answer the question of how fermented dairy products affect the development of cardio-metabolic diseases, 16 meta-analyses published in the last five years were identified and taken into consideration for this evaluation. In order to assess the associations of dairy products with cardio-metabolic diseases, we consequently focused on meta-analyses that combined the results of either observational studies [8,26,27,28,29,30,31,32,33,34,35,36,37,38,39,40,41] as well as two summarizing systematic reviews [42,43]. In addition, one meta-analysis of randomized clinical trials (RCTs) evaluating the effects of fermented dairy products on hypertension was published by Usinger et al. [44].

The major outcomes from this analysis are summarized in Table 2.

##### Cardiovascular Diseases (CVD)

Five meta-analyses have investigated the association between dairy product intake and CVD risk in the last five years [31,35,37,38,45].

The meta-analysis by Qin et al. [38] indicated that total dairy, but not yoghurt, may decrease the risk of CVD. Alexander et al. [31] indicated that total dairy, milk, yoghurt and cheese are not associated with reduced risk of CVD. Moderate evidence that higher intake of cheese is associated with a weak reduction of risk of CVD was reported by Chen et al. [32]. Moderate evidence for a weak reduction of risk of CVD for fermented dairy, but not for dairy, milk, cheese, or yoghurt was reported by Guo et al. [35]. The same authors also reported moderate evidence for a weak reduction of risk of mortality for fermented dairy, but not for dairy, milk, cheese, or yoghurt [35]. Finally, the meta-analysis by O’Sullivan et al. [37] indicated moderate evidence that total dairy, milk and cheese do not modify the risk of CVD mortality.

In their systematic review, Drouin-Chartier et al. [42] concluded that the association between the consumption of fermented dairy and CVD risk is based on very low-quality evidence and thus remains uncertain at this point (see Table S2).

Taken together, none of the meta-analyses reported a detrimental effect of dairy products, including all fermented dairy products investigated. A neutral effect of yoghurt was demonstrated in all four meta-analyses in which this product was investigated, whereas one out of five meta-analyses reported a beneficial effect of cheese consumption. On the other hand, the data from two meta-analyses on fermented dairy provided evidence for a beneficial effect of this product category. In conclusion, these meta-analyses provide weak evidence that fermented foods may have a beneficial effect on CVD, although the data remain weak and inconsistent.

##### Coronary Heart Disease (CHD)/Coronary Artery Disease (CAD)

Four meta-analyses have investigated the association between dairy products and CHD/CAD risk in the last five years [31,32,35,38]. Moderate evidence that higher intake of cheese is associated with a moderately reduced risk of CHD was reported by Chen et al. [32]. No evidence for a reduction of risk of CHD was found for dairy, milk, fermented dairy, cheese, or yoghurt [35]. Moderate evidence suggesting that cheese consumption, in particular, at higher servings, but not total dairy, milk or yoghurt, may be associated with a moderate reduction of risk of CHD was reported by Alexander et al. [31]. Finally, moderate evidence, reported by Qin et al. [38], suggests that cheese, but not total dairy or yoghurt, may moderately decrease the risk of CHD.

In their systematic review, Drouin-Chartier et al. [42] concluded that there is moderate but consistent evidence for a neutral association between yoghurt consumption and CAD risk. The same authors also concluded that the association between the consumption of fermented dairy and the risk of CAD remains uncertain because only evidence of insufficient quality is available (see Table S2).

Taken together, moderate evidence for a moderate reduction of risk of CHD was associated with the consumption of cheese in three out of four meta-analyses, whereas the other analyses, including yoghurt, milk and dairy, indicated a neutral effect.

##### Stroke

Four meta-analyses have investigated the association between dairy product consumption and stroke risk in the last five years [31,33,34,36,38]. The analysis by Alexander et al. [31] suggested that there is moderate evidence that cheese consumption, but not milk, may be associated with a moderate reduction in the risk of stroke, whereas total dairy consumption may be associated with a reduction in the risk of stroke. The analysis by de Goede et al. [33] indicated moderate evidence for a weak reduction in the risk of stroke with consumption of milk and >25 g/day cheese. Qin et al. [38] indicated that total dairy may decrease the risk of stroke and showed moderate evidence that cheese, but not yoghurt, may weakly decrease the risk of stroke. Also, Hu et al. [36] showed moderate evidence that total dairy, including fermented milk, but not milk or non-fermented milk, may moderately decrease the risk of stroke. Finally, the study by Hu et al. [36] suggested that there is moderate evidence that cheese intake may weakly decrease the risk of stroke.

In their systematic review, Drouin-Chartier et al. [42] suggested that there is moderate-quality evidence that the consumption of fermented dairy is associated with a reduced risk of stroke (see Table 2). They further concluded that the available meta-analysis on yoghurt has a relatively good quality score, further suggesting that yoghurt consumption is not associated with the risk of stroke; this was based on moderate-quality evidence.

Taken together, none of the dairy products, including fermented dairy products, are associated with a detrimental effect on stroke. Moderate evidence for a weak to moderate effect of fermented dairy products, in particular, cheese, is indicated by these meta-analyses, although these effects are inconsistently associated with the fermentation process—yoghurt was found to have a neutral effect when investigated in the product-specific study.

##### Hypertension

Two meta-analyses have investigated the association between dairy products and hypertension risk in the last five years [39,40]. In addition, one meta-analysis, integrating 15 randomized controlled trials (RCTs) evaluating the impact of fermented dairy products on hypertension, was published by Usinger et al. [44].

The study by Soedamah-Muthu et al. [40] suggested that total dairy and milk, but not yoghurt, total fermented dairy or cheese may moderately contribute to the prevention of hypertension. Also, the report by Ralston et al. [39] provided moderate evidence for a moderate effect of fluid dairy foods (including milk and yoghurt), but not cheese, on blood pressure in subjects with elevated blood pressure.

In the review of RCTs on the impact of fermented milk on hypertension, Usinger et al. [44] suggested a modest overall effect of fermented milk on blood pressure. However, the evidence was evaluated as weak, in light of the fact that an effect of fermented milk was found on systolic blood pressure (BP), but not on diastolic BP.

The included studies were of variable quality as well as heterogeneous, and the findings do not support the use of fermented milk as an anti-hypertensive treatment or as a lifestyle intervention to reduce blood pressure.

In their systematic review, Drouin-Chartier et al. [42] concluded that there is no significant association between the consumption of fermented dairy and the risk of hypertension. Of note, Drouin-Chartier et al. [42] commented on an additional published study on this topic [46], which reported an inverse association between the consumption of fermented dairy and the risk of hypertension. This study has an important weighting (*n* = 2340) relative to data from the meta-analysis by Soedamah-Muthu et al. [40] (*n* = 7641) and is likely to modify pooled risk estimates. In this context, Drouin-Chartier et al. [42] suggested that moderate-quality evidence supports a neutral association between the consumption of fermented dairy and the risk of hypertension, with the need for further studies on the topic to yield better quality evidence. Regarding yoghurt and the risk of hypertension, moderate-quality evidence was suggested by Drouin-Chartier et al. [42] that yoghurt consumption is not associated with the risk of hypertension (Table 2).

Taken together, none of the dairy products, including fermented dairy products, are associated with an increased risk of hypertension. Half of the studies reported weakly beneficial effects but the results are inconsistent.

##### Myocardial Infarction

No meta-analysis is available that summarizes studies characterizing the association of fermented dairy products and myocardial infarction risk.

##### Type 2 Diabetes Mellitus (T2DM)

Five meta-analyses have investigated the associations between fermented dairy products and T2DM risk in the last five years [8,26,27,28,41].

The meta-analysis by Gijsbers et al. [26] provided moderate evidence that the intake of dairy foods, yoghurt and fermented dairy, but not cheese or milk, moderately decreases T2DM risk. The study by Chen et al. [8] showed moderate evidence that higher intake of yoghurt is associated with a moderately-reduced risk of T2DM, whereas total dairy is not appreciably associated with the incidence of T2DM. Aune et al. [27] indicated that dairy products, but not milk, may be associated with a decrease in the risk of T2DM. Also, moderate evidence suggested that yoghurt at higher doses may moderately decrease the risk of T2DM. Finally, the same authors reported moderate evidence that cheese, but not cottage cheese, may weakly decrease the risk of T2DM, as well as weak evidence that fermented milk may moderately decrease the risk of T2DM. The fourth meta-analysis by Gao et al. [28] showed moderate evidence suggesting that the intake of dairy products, cheese and high doses of yoghurt, but not milk or fermented dairy, moderately decrease T2DM risk. Finally, Tong et al. [41] indicated that total dairy may reduce the risk of T2DM, whereas moderate evidence suggests that yoghurt, but not whole milk, may moderately reduce the risk of T2DM.

In their systematic review, Drouin-Chartier et al. [42] concluded that the consumption of fermented dairy does not appear to be associated with the risk of T2DM. This statement was based on moderate-quality evidence, because the three meta-analyses available relied on almost the same pools of prospective cohort studies (see Table 2). On the other hand, the same authors concluded that the five meta-analyses regarding the association between yoghurt intake and the risk of T2DM reported consistent results, suggesting that there is high-quality evidence that supports an inverse association between the intake of yoghurt and the risk of T2DM.

Taken together, these meta-analyses provide evidence for a positive impact of fermented dairy, in particular yoghurt, on T2DM risk.

##### Metabolic Syndrome (MetS)

One meta-analysis has investigated the association between dairy products and metabolic syndrome risk in the last five years [29].

This meta-analysis indicated that dairy intake may be inversely associated with the incidence and prevalence of metabolic syndrome. Also, weak evidence from cross-sectional studies suggests that dairy, milk and cheese, but not yoghurt, may moderately decrease the incidence of diabetes.

In their systematic review, Drouin-Chartier et al. [42] judged the quality of the evidence relating yoghurt intake to the incidence of MetS to be very low, and thus, the association remains uncertain.

Taken together, none of the dairy products, including fermented dairy products, are associated with an increased or a decreased risk of MetS.

##### Obesity

One meta-analysis has investigated the association between dairy products and metabolic obesity risk in the last five years [30].

This meta-analysis indicated, with weak to moderate evidence, that yoghurt consumption weakly decreases weight gain, waist circumference, risk of being overweight, and risk of abdominal obesity. The study also provided moderate evidence that cheese consumption weakly increases weight gain. In addition, dairy was negatively associated with weight gain, waist circumference, risk of being overweight and risk of abdominal obesity. Finally, milk consumption was negatively associated with waist circumference.

Taken together, yoghurt might be beneficial preventing obesity. However, no significant association for yoghurt consumption was observed for most of the endpoints related to obesity when comparing the highest versus the lowest categories of consumption. Further, the overall interpretation of the results is limited by heterogeneous risk estimates. The level of evidence for impacts of fermented dairy products on obesity risk is limited, and further studies are needed.

#### 3.1.2. Overview of Meta-Analyses Focusing on Interventional Studies Investigating Cardio-Metabolic Diseases

Drouin-Chartier et al. [43] conducted a comprehensive review of the impact of dairy foods, in particular, of dairy fat, on cardio-metabolic risk. This review included a range of randomized controlled trials (RCTs) as well as the meta-analyses of RCTs published by Benatar et al. [47] and de Goede et al. [48], and the systematic reviews of Turner et al. [49] and Labonté et al. [50]. These studies investigated LDL-cholesterol, HDL-cholesterol, fasting triglycerides, postprandial triglycerides, LDL particle size, apoB, non-HDL cholesterol, cholesterol ratios, inflammatory markers, insulin resistance, blood pressure, and vascular function.

Drouin-Chartier et al. [43] focused their analysis on the potentially detrimental effect of dairy fat on cardio-metabolic risk factors by concluding that there is no apparent risk of potential harmful effects of dairy consumption on a large set of cardio-metabolic variables. Among the products investigated, total dairy, milk, cheese, and yoghurt were discussed, providing additional information on the impact of fermented dairy products on cardio-metabolic health. The authors highlighted that the cholesterol-raising effects of saturated fatty acids (SFAs) are attenuated when provided in complex foods, such as milk, cheese, or yoghurt. Dairy food consumption has neither an impact on low-grade systemic inflammation, nor on insulin resistance or glucose and insulin homeostasis in the short term but may be beneficial in the long term. Furthermore, data from RCTs that have evaluated the impact of dairy consumption on either BP or vascular function are very consistent in showing mostly no effect.

In summary, an overview of the RCTs available on the impact of fermented dairy products on cardio-metabolic factors indicate that these products do not differentiate themselves from milk or total dairy in that their impact can be characterized as neutral.

#### 3.1.3. Meta-Analyses from Prospective Studies on Cancer

Nine meta-analyses, summarizing several cohort and case-control studies that were published in the last five years were used to characterize fermented dairy products in the evaluation of the influence of dairy products on cancer risk and cancer mortality [51,52,53,54,55,56,57,58,59]. The results of cohort studies and/or case-control studies were reported for several cancer types in two (colorectal, gastric) or one (lung, ovarian, pancreatic, prostate) meta-analyses.

##### Colorectal Cancer

Two meta-analyses from the last five years investigated the association between dairy products and colorectal cancer risk [51,52]. The first meta-analysis showed that milk and total dairy products are associated with a significant reduction in colon cancer risk, whereas cheese, yoghurt, fermented milk and fermented dairy have neutral effects [51]. Ralston et al. [52] later confirmed these findings by reporting a significant inverse association between the consumption of non-fermented dairy products and the risk of colorectal cancer, but no association between the consumption of fermented milk and cheese and colorectal cancer risk.

There is no evidence for a beneficial or detrimental effect of fermented dairy products on colorectal cancer. The potential beneficial effects of dairy products regarding colorectal cancer are thus unlikely to be attributed to the fermentation process.

##### Prostate Cancer

One meta-analysis investigated the association between dairy products and prostate cancer risk [53]. High intakes of dairy products, including cheese, but not milk and yoghurt, may increase total prostate cancer risk. This evidence was rated as limited in the WCRF/AICR 2007 report [60].

There is no evidence for a beneficial effect of fermented dairy products on prostate cancer. As different outcomes were reported for yoghurt and cheese intake, the weak evidence for a negative association of cheese consumption with prostate cancer is unlikely to be attributed to the fermentation process.

##### Pancreatic Cancer

One meta-analysis investigated the association between dairy products and pancreatic cancer risk that was published in the last five years [54]. Intakes of cheese, cottage cheese, yoghurt, as well as milk, were not associated with pancreatic cancer risk.

There is no evidence for a beneficial or detrimental effect of fermented dairy products on pancreatic cancer.

##### Gastric Cancer

Two meta-analyses investigated the association between dairy products and gastric cancer risk [55,56]. None of these analyses demonstrated a significant association between the intake of cheese and yoghurt, and gastric cancer risk. Of note, the results of cohort studies, but not case-control studies, suggested that total dairy intake might be related to the reduction of gastric cancer risk [57], whereas the results of case-control studies, but not cohort studies, provided weak evidence for an increased risk [55].

There is no evidence for a beneficial or detrimental effect of fermented dairy products on gastric cancer. The potential effects of dairy products on gastric cancer are thus unlikely to be attributed to the fermentation process.

##### Ovarian Cancer

One meta-analysis from the last five years, summarizing 19 cohort and case-control studies, investigated the associations between fermented dairy products and ovarian cancer risk [58]. This study concluded that milk and yoghurt intake has no association with an increased risk of ovarian cancer.

There is no evidence for a beneficial or detrimental effect of fermented dairy products on ovarian cancer.

##### Lung Cancer

One meta-analysis from the last five years has summarized cohort and case-control studies in order to investigate the associations between fermented dairy products and lung cancer risk [59]. Weak evidence from two cohort studies was available for a protective effect of cheese, but this effect was not found in the overall analysis of all studies that included eight case-control studies. In addition, no effects were observed for dairy, milk and yoghurt.

Taken together, there is no evidence for a beneficial or detrimental effect of fermented dairy products on lung cancer.

##### Other Cancers

No meta-analysis is available that summarizes the impact of dairy products or fermented dairy products on other types of cancer. Also, to our knowledge, no individual study has been published focusing on the effects of fermented dairy product intake on additional types of cancer whose results would justify a critical appraisal in this report.

##### Summary of Studies Involving Cancer

In their review, Thorning et al. [61] concluded that, according to the World Cancer Research Fund reports and the latest meta-analyses, (i) consumption of milk and dairy products probably protects against colorectal, bladder, gastric and breast cancers, (ii) dairy intake does not seem to be associated with risk of pancreatic, ovarian or lung cancer; and (iii) the evidence for prostate cancer risk is inconsistent.

### 3.2. Meta-Analyses of Randomized Controlled Trials with Probiotics

Eight RTCs have evaluated the impact of probiotics on cardio-metabolic risk factors in the last five years [62,63,64,65,66,67,68,69]. These studies are completed by the review by Barrett et al. [70] investigating randomized and cluster-randomized trials for the impact of probiotics on the risk of gestational diabetes. The factors investigated in the meta-analyses were body mass index (BMI), body weight and waist circumference, fasting glucose, HbA1c, HOMA-IR, fasting insulin, total cholesterol, LDL-cholesterol and HDL-cholesterol, triglycerides, malondialdehydes, as well as systolic and diastolic blood pressure.

However, as already mentioned above, after reviewing the results, we decided to exclude probiotics because not enough information was available to differentiate between the different strains or to find any clear association between specific strains and non-communicable diseases.

### 3.3. Fermented Foods of Plant Origin

Fermented plant products comprise a large variety of foods, including, for example, coffee, chocolate, sourdough, soybeans, cabbage, olives and alcoholic beverages, such as wine or beer. Similar to dairy products, plant foods are subjected to diverse strains of the *lactobacillus* and *bifidobacterium* genera [71] or, in the cases of wine, beer and sourdough, to *saccharomyces cerevisiae* and other yeast species or to a combination of these [72]. In general, fermented plant products are known for their contents of secondary phytochemicals, bioactive peptides or other compounds that may reduce blood pressure, fasting and postprandial blood glucose and insulin concentrations and which may act as natural antioxidants as well as immune modulators [73].

In fact, hardly any studies or meta-analyses have been published regarding the effects of plant-derived fermented foods on human health and disease. Most of the existing studies were performed in in vitro or sometimes, animal models, and very often, researchers tested single food compounds rather than the whole product. This needs to be considered when interpreting data as the concentrations of the phytochemicals, for example, vary with varying fermentation times leading to different metabolic responses.

Hardly any information is available in terms of the risks of developing diet-related chronic diseases and the consumption of Swiss-typical fermented foods of plant origin.

However, in Switzerland, there is not one specific fermented food of plant origin, except for wine, beer and coffee, being traditionally and regularly consumed, such as kimchi in Korea or fermented soy sauce in Asia. However, the sum of all fermented products, including dairy products, is estimated to comprise approximately one third of the daily diet (globally) [74].

Hereafter, we focus on certain fermented foods of plant origin with relevant intake frequencies amongst the Swiss population and their effects on health and disease.

#### 3.3.1. Coffee

Coffee, and its effects on health and disease, have been extensively studied in the past in comparison to other fermented foods of plant origin. The meta-analyses performed in the past have produced controversial results in regard to positive, neutral or inverse associations with CVD incidence, CVD risk factors and CVD mortality. Four cups of coffee per day were found to be the optimal amount of coffee for inverse associations with death, CVD and CVD-related risk factors [75,76,77,78,79,80]. The highest consumption levels of coffee tend to be either protective against or not associated with other diet-related chronic disease risks. In particular, the highest consumption level may significantly reduce the risks of type 2 diabetes and cancer (in particular liver, esophagus, oral cavity, colorectum, thyroid, endometrium, colon, pancreas, and breast) by a maximum of 24% and 50%, respectively. However, positive associations have also been observed as the highest levels of coffee consumption might significantly increase the risks of obesity, other cancers (prostate, urinary tract, bladder), and high BP [81].

#### 3.3.2. Wine and Beer

The association between wine consumption and the risk of CVD and cancer is controversial, ranging from protective, to neutral, to deleterious effects; this association is strongly dependent on the quantities consumed. High levels of consumption (270 mL/day) were associated with a reduced risk of CVD (by a maximum of 45%) and cancers (by a maximum of 60%, in particular, esophagus, kidney and lung), as reported by Fardet and Boirie [81]. However, very high wine consumption (950–1985 mL/day) was associated with a maximum of +76% risk for CVD and +630% risk for head and neck cancers. Each increase of 10g alcohol from wine per day was associated with a 5% higher risk of breast cancer [81].

Beer consumption was inversely associated with CVD risk and CVD related risk markers, although the associations were less strong in comparison to wine [82,83].

#### 3.3.3. Sauerkraut

Clinical data about the effects of sauerkraut on the human organism, health and disease are scarce. There is knowledge concerning particular compounds in sauerkraut and their impacts on diseases; however, a literature search revealed mostly cell line or rat experiments with very limited conclusions for humans. Nonetheless, those study outcomes are promising in terms of positive effects of sauerkraut in humans, e.g., as a vehicle of probiotics, in cancer prevention, its free radical scavenging and anti-inflammatory potential. However, evidence for these implications is low [84].

#### 3.3.4. Fermented Olives

Fermented olives are a rather unexplored fermented food and a source of potentially beneficial compounds, including microbial strains and bioactive components. It is assumed that fermented olives display anti-cancer, antioxidant, anti-inflammatory and anti-bacterial properties, with oleic acid, antioxidants, phenolic compounds and lactic acid bacteria being proposed as the strongest contributors to these effects [85]. However, most of the studies investigating the effects of fermented olives have been conducted in vitro. Fermented olives are an important part of the Mediterranean diet that is associated with a low incidence of chronic diseases like T2DM or CVD. However, the actual extent of their contribution to the prevention of non-communicable diseases cannot be determined due to missing clinical data [86]. Of note, the consumption of this food does not occur in public health-relevant amounts in Switzerland.

## 4. Discussion

The first clear outcomes of this report are that (i) the consumption of fermented foods is not associated with negative risks to humans with regard to the discussed clinical indications, i.e., cardio-metabolic health and cancer; and (ii) the majority of the results are associated with neutral effects.

Most, if not all, of the clinical data summarized in meta-analyses have been obtained with fermented products as part of total dairy intake (mostly prospective cohort studies), with other sources of fermented foods being much less investigated so that studies of sufficient quality are not available to perform meta-analyses.

Generally, the associations of fermented dairy with cardio-metabolic diseases and cancer comply with those of dairy products—this association being neutral for cancer and weakly beneficial, though inconsistent, for specific aspects of cardio-metabolic health. The strongest evidence for a beneficial effect of fermented dairy products was found for the effects of yoghurt on risk factors associated with type 2 diabetes. However, for all combinations of indications and dairy products reviewed in this report, the heterogeneity in the design of the studies as well as in the products being investigated, impedes a definitive assessment of the association between product consumption and health. In that context, it is unclear, and rather unlikely that additional studies will provide more robust answers to these questions. The above statement is not only restricted to the evaluation of fermented dairy products but is also relevant to other fermented foods.

The literature provides some information on the bioactivity and mechanisms mediating the impact of fermented foods in health and disease prevention, in particular, in regard to dairy products [87]. The health benefits associated with the fermentation process may be the result of direct interactions between the ingested live microorganisms and the host (probiotic effect), or indirectly, through ingestion of microbial metabolites and products of fermentation (biogenic effect) [88]. During the fermentation process, a wide range of peptides are released by proteolysis from caseins and whey proteins, some of them with bioactive effects, like blood pressure lowering or thrombin inhibition [89]. Furthermore, a cholesterol lowering effect has been described in relation to exopolysaccharide-producing bacteria during fermentation, which results in increased synthesis of bile acids from cholesterol, decreasing the circulating level of cholesterol [90]. Other proposed mechanisms are the assimilation of cholesterol by bacterial cells, also resulting in reduced absorption of exogenous cholesterol in the small intestine, or the conversion of cholesterol to coprostanol [91]. Finally, bacterial cultures used for fermentation are known to synthesize vitamins, like folate, riboflavin, vitamin B12, or vitamin K2 (menaquinones), which are all involved in pathways important for cardiovascular health [21].

More specifically, several mechanisms associated with the fermentation process may contribute to the inverse association between yoghurt intake and risk of T2D. On one hand, probiotic bacteria have been shown to improve lipid profiles and antioxidant status in T2D patients [92] and have beneficial effects on cholesterol levels [93]. On the other hand, the accumulation of insulinotrophic peptides and amino acids [94,95] and the microbial synthesis of vitamins like menaquinones [8] may improve insulin sensitivity. For the negative association between cheese intake and stroke, the evidence points more towards a consequence of technology, rather than fermentation, as cheese making significantly enriches calcium and magnesium, which have been associated with reduced risk of stroke [96,97].

Thus, the literature is characterized by a wealth of data on the bioactivity of fermented foods, including metabolites and bacteria, which might beneficially contribute to a large range of physiological properties. However, the mechanistic data is not strongly linked to the results of the studies reviewed by meta-analyses. This gap can be explained by a range of factors, including the complexity of the investigated food–health interactions, the quality of the studies, and the real magnitude of the health benefits offered by these products. Further research should specifically integrate the impact of fermentation on risk factors in the study design, including the statistical approach. Such an approach may indeed provide a better indication of the impact of fermented foods on health.

## 5. Conclusions

Our report indicates that the consumption of fermented foods in the context of particular indications, such as yoghurt intake and diabetes or cheese intake and stroke, can only be recommended on the basis of weak and inconsistent evidence. Intervention studies on fermented foods with appropriate controls, study design and statistical methods, would ameliorate the evidence rating. Ideally, future meta-analyses should be able to summarize data from a range of RCTs evaluated in a cross-over study design, e.g., the effects of yoghurt, compared to unfermented milk in interventions of several weeks in prediabetic or diabetic subjects.

## Figures and Tables

**Table 1:** European Union member states explicitly mentioning yoghurt and probiotics in their nutrition guidelines or recommendations (taken from [23]).

	Dietary Guidelines Given by	Probiotic Genera/Species Mentioned in Dietary Guidelines	Advantages of Eating Probiotic Yoghurt Mentioned in Guidelines
Estonia	Terviseamet Health Board (Governmental Health Authorities)	*Lactobacillus acidophilus*; *Bifidobacterium* spp.	Help to maintain healthy human intestinal microflora
Germany	Federal Ministry of Food, Agriculture and Consumer Protection	-	Probiotic products contain special lactic acid bacteria that colonize the intestine and can stimulate the digestive functions from there
Italy	Expert group mandated by the Ministry of Health	*Lactobacillus* spp.	The bacterial flora in yoghurt metabolizes lactose, thus avoiding complaints about lactose intolerance; live lactobacilli exert beneficial effects on the organoleptic characteristics of food as well as on the gut (probiotic effect)
Poland	National Food and Nutrition Institute	*Lactobacillus* spp.; *Bifidobacterium* spp.	Modulate balance of intestinal bacterial flora and may provide beneficial health effects, such as regression of acute diarrhea in children, regression of inflammatory bowel diseases (ulcerative colitis and Crohn’s disease) as well as symptoms of irritable bowel syndrome, helping in the proper working of the immune system, and reducing the incidence of allergies in children
Spain	Ministry of Health, Social Services and Equality	*L. acidophilus*; *Lactobacillus casei*; *Lactobacillus reuteri*; *Lactobacillus plantarum*; *Bifidobacterium* spp.	Have immunological and protective properties in the gut

**Table 2:** Evaluation of the impact of dairy products on cardio-metabolic factors and diseases (intervention studies and prospective studies). The table was adapted from [42,43]. The evaluation method is presented in Table S1.

	Total Dairy	Milk	Cheese	Yoghurt
Prospective studies [42]				
CVD	Neutral	Uncertain	Neutral	Neutral
CAD/CHD	Neutral	Neutral	Neutral	Neutral
Stroke	Favorable	Neutral	Favorable	Neutral
Hypertension	Favorable	Favorable	Neutral	Neutral
MetS	Favorable	Favorable	Uncertain	Uncertain
T2DM	Favorable	Neutral	Favorable	Favorable
Interventional studies RCTs [43]				
LDL cholesterol	No effect	No effect	No effect	No effect
HDL cholesterol	No effect	Uncertain	Uncertain	No effect
Fasting TGs	No effect	Uncertain	No effect	No effect
Postprandial TGs	Undetermined	No effect	No effect	Undetermined
LDL size	Undetermined	No effect	Undetermined	Undetermined
apoB	Undetermined	No effect	No effect	Undetermined
Non-HDL cholesterol	Undetermined	Undetermined	Undetermined	Undetermined
Cholesterol ratios	Undetermined	No effect	No effect	Reduced
Inflammation	No effect	No effect	Undetermined	No effect
Insulin resistance	Uncertain	No effect	No effect	No effect
Blood pressure	No effect	No effect	Undetermined	No effect
Vascular function	No effect	No effect	Undetermined	No effect

apoB: apolipoprotein B; CAD: coronary artery disease; CHD: coronary heart disease; CVD: cardiovascular disease; HDL: high-density lipoprotein; LDL: low-density lipoprotein; MetS: metabolic syndrome; T2DM: Diabetes mellitus type 2; TG: triglyceride.

## Supplementary Materials

The following are available online at http://www.mdpi.com/2072-6643/10/4/448/s1. Table S1: Method used to evaluate the strength of the effect and the quality of the evidence provided by the literature; Table S2: Summary of the evidence for the impact of dairy products on cardiometabolic diseases; Table S3: Summary of the evidence for the impact of dairy products on cancer; Table S4: Summary of the evidence for the impact of probiotics on cardiometabolic diseases.
